# A Wake-Up Call: Equity, Inequality and Covid-19 Emergency Remote Teaching and Learning

**DOI:** 10.1007/s42438-020-00187-4

**Published:** 2020-09-23

**Authors:** Laura Czerniewicz, Najma Agherdien, Johan Badenhorst, Dina Belluigi, Tracey Chambers, Muntuwenkosi Chili, Magriet de Villiers, Alan Felix, Daniela Gachago, Craig Gokhale, Eunice Ivala, Neil Kramm, Matete Madiba, Gitanjali Mistri, Emmanuel Mgqwashu, Nicola Pallitt, Paul Prinsloo, Kelly Solomon, Sonja Strydom, Mike Swanepoel, Faiq Waghid, Gerrit Wissing

**Affiliations:** 1 University of Cape Town, Cape Town, 7700, South Africa; 2 University of Witwatersrand, Johannesburg, 2000, South Africa; 3 Central University of Technology, Bloemfontein, 9301, South Africa; 4 Queen’s University Belfast, Belfast, BT7 1NN, UK; 5 Nelson Mandela University, Port Elizabeth, 6019, South Africa; 6 Rhodes University, Grahamstown, 6139, South Africa; 7 Mangosuthu University of Technology, Durban, 4031, South Africa; 8 Stellenbosch University, Stellenbosch, 7600, South Africa; 9 Sol Plaatjie University, Kimberley, 8301, South Africa; 10 Cape Peninsula University of Technology, Cape Town, 7535, South Africa; 11 University of Zululand, Richards Bay, 3900, South Africa; 12 University of Pretoria, Pretoria, 0002, South Africa; 13 Durban University of Technology, Durban, 4001, South Africa; 14 North West University, Vanderbijlpark, 2520, South Africa; 15 University of South Africa, Pretoria, 0002, South Africa

**Keywords:** Equity, Inequality, Covid-19, Pandemic, Teaching, Learning, Higher education, South Africa

## Abstract

Produced from experiences at the outset of the intense times when Covid-19 lockdown restrictions began in March 2020, this collaborative paper offers the collective reflections and analysis of a group of teaching and learning and Higher Education (HE) scholars from a diverse 15 of the 26 South African public universities. In the form of a theorised narrative insistent on foregrounding personal voices, it presents a snapshot of the pandemic addressing the following question: what does the ‘pivot online’ to Emergency Remote Teaching and Learning (ERTL), forced into urgent existence by the Covid-19 pandemic, mean for equity considerations in teaching and learning in HE? Drawing on the work of Therborn (2009: 20–32; 2012: 579–589; 2013; 2020) the reflections consider the forms of inequality - vital, resource and existential - exposed in higher education. Drawing on the work of Tronto (1993; 2015; White and Tronto 2004) the paper shows the networks of care which were formed as a counter to the systemic failures of the sector at the onset of the pandemic.

## Introduction

What does the ‘pivot online’ to Emergency Remote Teaching and Learning (ERTL), forced into urgent existence by the Covid-19 pandemic, mean for equity considerations in teaching and learning in Higher Education (HE)? What are the risks in environments fraught with inequality? In what ways do emergency responces to the Covid-19 pandemic provide opportunities, which can leverage an equity agenda in the medium to long term, in the post-pandemic future?

This piece offers the collective reflections of a group of teaching and learning and HE scholars (i.e., academics, educational technology specialists and academic staff developers) produced from our experiences at the outset of the intense times when lockdown restrictions began in early 2020. As such, this article presents a rare snapshot of a particular time.^1^ The authors of this paper are from 15 of the 26 South African public universities, forged from pre-pandemic networks,^2^ who had already been supporting teaching and learning in a blended future, all of whom found themselves on the educational ‘frontline’ overnight. The battle has been to #savetheacademicyear, #savelives and to save life chances as the academic project has teetered beyond its already frail condition. While stories fill our inboxes and social networks, there is an expressed hope that, somehow, this ‘wake-up’ call will result, post-pandemic, in reshaping the intersections of equity, inequality and teaching online for the better.

Our focus in this piece is on how issues of equity and inequality have played out in the ‘pivot’ to remote teaching and learning. Our interest is in acknowledging the dangers and responses to risk. Our concern is for the future post-pandemic.

Of the 26 South African public universities, 25 are residential institutions, which have been governed by regulations that did not allow distance education until 2014. The single (400 k + students) distance education provider was impacted by restrictions too, as it had been neither distributed for staff nor fully online. The ERTL shift would inevitably play out differently across the HE sector given the variety of institutional types categorised in different ways: rural and urban, old and new, Historically Advantaged Institutions (HAIs) and Historically Disadvantaged Institutions (HDIs), and ‘research-intensive’, ‘comprehensive’ and ‘universities of technology’.

The pandemic sees different forms of inequality coexisting. Drawing on Therborn’s (2009; 2012; 2013) three categories of inequality, it is possible to capture the contemporaneous inequalities of the early days of ERTL.

Vital inequality refers to ‘life chances’, and indeed to survival rates. Even prior to the pandemic, studies indicated that not only do educated people generally live longer (Meara, Richards, and Cutler 2008), their parents do too (Friedman and Mare 2014; Ingraham 2014). In an HE sector infiltrated by Covid-19, confronting vital inequality is inescapable—by keeping residential universities open with students and staff in physical classrooms, lives and health are literally at risk. Yet, given that the ‘pivot online’ is experienced unevenly by universities across the sector, and students within the sector, the life chances of certain students in particular are cruelly diminished.

In South African HE, resource inequalities in recent years have been at the forefront under the guise of #FeesMustFall, while during the pandemic, they have been the unsurprising focus of attention because of immediately obvious digital divides. However, while resource inequalities manifest in this period most overtly through material divides, also essential are the range of capitals needed to negotiate and survive the crisis. Unequally distributed, ‘human actors have very different resources to draw upon’ (Therborn 2012). While illustrated with reference to the role of social capital and inequality of rewards or outcomes in Therborn’s definition, such capitals are elaborated on to include other forms of cultural and affective capital too.

Existential inequality is described as the ‘denial of equal recognition and respect and is a potent source of humiliations’ (Therborn 2020: 580). Acknowledged as ‘not only blatant discrimination’ (Therborn 2020: 580), existential inequality refers then to dignity, autonomy and representativity which had been the focus of the recent #RhodesMustFall protests and which continue to underpin pandemic-mediated lived experiences through the politics of misrecognition (Fraser 2007).

These three types of inequality are shaped by and are in the nexus of mutually constitutive factors such as gender, culture, race, class and geopolitical context. While ‘we are all mortal and physically vulnerable’, it is necessary to acknowledge that ‘in some sense our life-tree is decided by some inscrutable lottery’ and that our life chances ‘are distributed with clearly visible social patterns’ (Therborn 2012: 80). These three kinds of inequality interact with and influence each other. However, it is useful to distinguish between them because, in addition to having different types of effects on people, the different kinds of inequality have different trajectories in different periods—which means that they are governed by different causal mechanisms (Therborn 2009).

Covid-19 has threatened our ‘world [which] includes our bodies, ourselves, and our environment, all of which we seek to interweave in a complex, life-sustaining web’ (see Fisher and Tronto 1990: 40). How to ‘heal’ this world is a practice that Tronto would define as an ethic of care: ‘a species activity that includes everything that we do to maintain, continue, and repair our “world” so that we can live in it as well as possible’. How we in education have attempted to ameliorate the challenges we and our students have encountered have taken the form of acts of care. Yet, every caring act occurs in a larger political context that reflects a given society’s values, laws, customs and institutions (Tronto 2015: 10).

Underpinned by the concepts of inequality and ethic of care, in the following sections, we offer a thematic analysis from academics’ and professionals’ written reflections in response to these questions: how have issues of equity and inequality played out in the ‘pivot’ to remote teaching and learning, as well as to the associated systems and processes? What were the risks and the related responses to these risks? What concerns for the future post-pandemic remain?

From the analysis, nine themes emerged that provide insight into the current status of responses to Covid-19 with regard to teaching and learning through an equity lens. Written individually at first, these reflections were made in March 2020, at the outset of ERTL—in the middle of the first semester of the local academic calendar. Original extracts from our written reflections form part of the narrative in italics; these are our individual musings based on the stories we experienced directly, saw, heard, or encountered in our work inside our universities respectively. We do not offer a sector-wide perspective (indeed at the time of writing some of the country’s institutions had yet to start ERTL) nor speak on behalf of our institutions. However, as a collective of teaching and learning actors, these experiences draw a distinct picture of the diverse and highly unequal positions in which we find ourselves. We decided against labelling comments in order to preserve complete anonymity, agreed at the outset to enable uncensored reflections. Narratives were first extracted by one author, and shared with all participants, who, to varying degrees, commented and edited. Some of us changed our words in relation to the synthesis, some engaged specifically with theory. This process of collective meaning making, or ‘collective reflection’, refers to any type of dialogue among practitioners or researchers, that is aimed at making meaning from diverse experiences and perspectives. It consists of both individual self-reflection activities and dialogical encounters through engaging with each others’ written work (De Lawter and Sosin 2000). Often used in participatory research methodologies, collective reflection emphasises the social nature of meaning construction and affirms the authentic expression of personal knowledge. Recent literature has pointed to a move from individual to collective reflection, affected by trends in professional practice characterised by a collective rather than individual focus; multidisciplinary or transdisciplinary practices; as well as an increasing emphasis on co-production in practice (Foong, Nor, and Nolan 2018).

## On Not Unseeing: Inequalities Made Visible

The current crisis has made it impossible not to recognise the historical, geospatial, economic inequalities of the country and the world students live in. In a certain sense, the pandemic, and the pivoting to online made visible, the invisible, or ignored manifestations and mechanisms of inequality. The HE sector in South Africa is characterised by low participation and high attrition, with students from higher socio-economic groups more likely to be eligible for university entrance (Van der Berg 2016), and white students more likely than black students to succeed (Council on Higher Education 2014: 63). With university success improving life chances, the immediate fear is that the shift to ERTL will undermine prospects of success.

Especially in residential universities with onsite residences, computer laboratories and Wi-Fi, it is possible for existing differences of background to be covert. Across the nation, *the pandemic revealed historic (and mostly forgotten) fault lines, and as silence settled down upon buzzing cities and communities and we all came to a standstill, we were forced to hear the tectonic layers pushing and shoving against one another, tectonic layers of intergenerational inequalities, unheard and ignored for too long.*

This observation reverberates, *in/equity issues have been with us [for] as long as I can remember so now everyone understands what we mean by inequality. Those who have been pretending not to know and those who really didn’t know*. Arguably, nothing is new. *A number of barriers have been mentioned as being inserted by the current situation when, in fact, those barriers existed before and are only being made visible by the current situation. Where socio-economic inequality exists, it existed before. Where language barriers exist, they existed before.*

Of course, this is not the first time that these issues have been brought to light: *the Covid-19 pandemic and the resultant lockdown has not uncovered anything that we did not already know about our students, but as with #FeesMustFall, it has placed the challenges of inequity in stark relief. Indeed, the #FeesMustFall shutdown should have served as a rehearsal for the lockdown created by the pandemic.* Universities are now *provided with opportunities to rethink strategies to enhance the success of all students, especially those who are facing socio-economic challenges. This has served as a wake-up call.*

Yet, while the sounds of the tectonic layers of inequality were and continue to be heard in HE, what did change *is that institutions that despised remote and distance education, and looked down on online learning suddenly embraced online, remote and distance learning as if they were long lost cousins, albeit from the poorer side of the family.*

Although much has been made of the technology and the mode during this time, what has been educationally more critical is how the speedy move to ERTL exposed poor pedagogical practices, in particular *practices which were insufficiently designed for actual students in asymmetric lives rather than ideal students.* Certainly, *where a technology-first approach to learning design exists, it existed before, where difficulties exist with assessment now, many* *of those difficulties arose through poor assessment practices practised while on campus.* And *where well-designed interactive learning interactions—however documented—are absent, they were absent before*. Even as we acknowledge that ERTL is not the same as online teaching (Hodges, Moore, Lockee, Trust and Bond 2020), it has become clear that *a great measure of the challenges around students now not being able to continue learning meaningfully, has far more to do with preexisting poor curriculation-for-context practices.*

This enforced visibility has made the covert overt: *the lockdown has forced us to look much closer to where our students are, where they are positioned, what resources they have, what opportunities to engage in teaching and learning. And we cannot unsee these differences, whether on or off-campus.* Enforced visibility places a renewed focus on contextual realities and students’ lived experiences. It forces us to critically consider aspects conducive for teaching and learning without contributing to new forms of digital exclusion.

Referring back to Therborn (2009), the pandemic and ERTL have revealed many of the causal mechanisms perpetuating inequality such as allowing students who did not have sustainable and affordable access to further fall behind (distantiation) and being excluded. Moreover, an already hierarchised teaching and learning context became further hierarchised. Students and staff are facing exploitation, not only through the colonisation of teaching and learning spaces by commercial providers to which many institutions have turned, but also through the increasing data gaze of educational technology and erosion of privacy and data sovereignty that were of concern prior to the pivot online (Beer 2018; Kukutai and Taylor 2016).

## Embedded in Context

The sudden shift to ERTL takes place within existing contexts, histories and cultures. The pandemic hit an already unsettled sector, where physical closures of campuses in the form of ‘shutdowns’ had been widespread. As Therborn (2009; 2013) points out, drawing from a long history of intersectionality scholars (Crenshaw 1989; hooks 1994; 2000a; 2000b; Yuval-Davis 2006), inequality and the causal mechanisms perpetuating inequality should be understood and mapped in context as inequalities are shaped by and in the nexus of mutually constitutive factors such as gender, culture, race, geopolitical context, etc. While the move to ERTL therefore impacted the whole (higher) education sector, the impacts differed between rural and urban, old and new, HAIs and HDIs, and ‘research-intensive’, ‘comprehensive’ and ‘universities of technology’.

Some universities postponed the shift to ERTL later in the year, because of existing instability from interactional dynamics. In one case, *the delay was partly due to the fact that our university had already been closed before the lockdown because of student protest.* Elsewhere, *there were very few classes taking place due to student unrest and some of those students have not even [been] registered for all of their subjects. Furthermore, only a few days of real classes happened on that campus. These students are severely disadvantaged.* Thus, preparedness was determined by existing conditions across different university physical locations, where on-the-ground events were inextricably historical.

In other contexts, a number of factors contributed to students’ capacity to move to remote learning as *students’ computer skills training did not happen because of strikes at the beginning of the year and obviously of Covid-19... most of them come from schools where they were not introduced to computers... and one or two online sessions will not necessarily be sufficient.* Covid-19 in this case added an additional layer to historical disadvantage and unrest.

The dramatic shift took place within existing organisational cultures. In some cases, *this lockdown has exposed the alienating practices we are exposed to.* Within universities, there is often distrust of university management, with the feeling that concern for students has not been the priority, but rather *as was the case during #FeesMustFall, we wanted to protect our reputation first.* There is *distrust between students regarding the uni’s approach—organised bodies representing the students calling them to reoccupy residence halls as [they are] worried no one had their [students’] interests at heart and their possessions were being pilfered... [and a] toxic work environment simply means very polemic and ungenerous relations between staff.* The doubt about the intentions of those in leadership and their relationship to student voice harks back to previous shutdowns: *it is surprising that management in some universities have not learned from the #FeesMustFall era.* *There are universities that are failing to convince their students and student leaders about their commitment towards making sure that no student will be left behind.* Indeed, expectations from some of us were so low that evidence of high-level commitment was unexpected: *the total commitment of our senior executives to do, within reason, whatever is necessary to ‘make this work’ for everyone still surprised me.*

The pandemic also hit the sector in a decade where student activism in South Africa has been reignited since the political activism within the education sector during apartheid (Diseko 1992). The years of active student protest and campus disruption since 2015 were fresh in everyone’s minds. *Where students have doubts about the commitment [to addressing inequities], they are likely to oppose remote online learning as a solution to the* *problems caused by the pandemic.* Indeed, student protests did continue, mediated, ironically, by the digital spaces that have enabled much student activism in the country (Maluleke and Moyer 2020) and in other developing contexts (Sharma 2020) and through hashtags such as #stayoffline. These responses resonated with the previous shutdowns, which for many had been a distressing period from which they were still reeling, and which, in some cases, had continued without pause into the start of the pandemic.

Existing institutional cultures have been both shaped and unsettled by ERTL, in terms of the roles of senior management as well as non-academic staff, given that *lecturers get some recognition for the burden of shifting online, but truly, it is the invisible administrative staff who are keeping the ship afloat.* It becomes clearer how institutional cultures and hierarchies are themselves causal mechanisms of inequality (Therborn 2009). Academics who often see themselves at the top of the institutional prestige hierarchy were faced with the realisation that ICT, eLearning support, planning units, student and academic support, security, and a range of others ‘lower’ in the institutional hierarchy, were essential in making ERTL happen.

## The ‘Least-Worst Option’: Multipronged, Multimodal Strategies

The Minister of HE announced in April 2020 that South African universities would need to move to ‘multi-modal remote learning systems including digital, analogue and physical delivery of learning materials in order to provide a reasonable level of academic support to all our students at all institutions in order to save the academic year’ (Timeslive 2020). Aptly described as *the least-worst option*, this option was weighed against the more negative alternative to close all universities until the pandemic was officially over and all students as could return to campus. This alternative of closure was considered too high for the sector as a whole, for specific institutions and for individual students *the risk of complete closure would be that some institutions would never re-open and many students would fall out of the system altogether.* With low participation rates, institutional closures of the few public universities would have a larger impact on the access agenda of the country (at 22% South Africa’s tertiary enrolment rate is well below the global average of 38%) (World Bank 2017). Variegated plans and options are necessary because, *without such a multipronged strategy, inequities will occur because of students who have no access to devices and connectivity. This would be the case in institutions that will choose to only teach remotely using technology.* Early on, we were aware that it may not be possible to reach all students remotely, and doing so *may cause more stress and limit those who have access when trying to develop a model that would engage everyone.*

As noted in the first theme, *choosing remote online learning as an emergency measure to deal with the impact of the pandemic has served as a painful reminder of all the inequalities that the system has managed to mask.* Such a multi-pronged strategy is *almost an impossible task* - one amplified by the #stayoffline movement which called for all students to not study online until the university sector ensured that all students ‘are not left behind’. Despite this, coupled with initiatives to develop alternative scenarios, the HE Minister’s statement that ‘every effort is now being made to put in place multiple and flexible methods of teaching and learning to support all our institutions and all our students’ (Timeslive 2020) *has seen this quest for the seemingly impossible. Indeed, we were surprised at the lack of debate as to whether one should or should not continue teaching remotely* and noted that *the complete lack of consideration of that option was interesting* in itself. Although there was some resistance and criticality expressed in response to the nationally decreed multimodal strategy (Naidu 2020), and the underlying conditions had not changed since the previous student-led shutdowns, these protests did not gain the kind of traction which had occurred earlier.

We observed that it was *a watershed moment when the question changed from* *Should we go online? to How should we go online?* *A different, pragmatic formulation of approaching social justice finally seems to be emerging. Rather than risking doing nothing, so that no-one is disadvantaged, the response is that we do whatever we can to address barriers to access in the moment, and where some barriers simply cannot be addressed in the moment, we have a campus-based ‘second opportunity’. Such a response is at the heart of the great strides that are being made towards addressing barriers... Barriers aren’t being removed entirely, but significant progress is made. Even more heartening is that… long-term solutions to matters challenging digital equality are being sought actively and addressed where possible. A certain shift in mindset has occurred.* In short, this shift in mindset pertains to format, form, time, pedagogy and place.

A multimodal approach requires course content to be *highly accessible through multiple media formats for students to choose that which is most conducive to learning within their context*. From an equity perspective, it is relevant that these modes are not all digital. For instance, *in areas where there is no connectivity, students will receive teaching and learning material through post. The multipronged approach has seen temporal adjustments to timetables, dates of delivery, and forms of assessment.* Thus, *forms of teaching provision have had to become multidimensional and flexible; at the outset of the pandemic, it is unknown whether these attempts have been effective and for whom.* Planning for multiple scenarios was done under duress at great speed *as the unpredictable nature of the pandemic made informed decision-making difficult and decisions were frequently presented using varying scenarios, heightening levels of anxiety.* To make ERTL work *in such a way that ‘no student is left behind’ requires nuanced ways of provision. So, given the limited time and resources, experience will reveal if the multimodal response may unwittingly be inserting an additional barrier, or [whether] we will be able to timeously ensure equity in the design, provision and support of the various learning modalities.*

The move to ERTL and the speed at which decisions had to be made, meant a move back to basics, and many of us emphasised the need to ‘keep it simple’. The next section presents a cursory overview of what such a move entailed.

## The Basics: Making a Plan

All universities, even the most historically advantaged and well-resourced, have had to consider issues of resourcing at the individual level of all the people within their institutions. This includes the reality that many students and staff either lack or have inadequate devices, data and connectivity to enable or undertake academic activities.

Pre-pandemic, a part of the university education was understood to be about preparing students for living in a society intrinsically digitally mediated. At the early stages of the pandemic, the infrastructure became essential, and thus addressing material needs became non-negotiable. With only a 53% Internet penetration rate and amongst the highest mobile phone data costs in Africa (Chinembiri 2020), South Africa has a low infrastructural starting point with deep digital divides. Our narratives revealed a pattern of problem-and-solution: with each problem being solved in some way, another appeared. This is how digital inequalities morph into new forms.

The first problem is that the infrastructure for digital education relies on electrical power. However, structural inadequacy to address the demand for power in addition to maintaining consistent and reliable provision has dogged the country since 2007. The extensive scheduled electricity cuts (‘load shedding’) practiced by the state-owned provider were mitigated against in campus environments. However, *having a high-level LMS has not helped the majority that still studies over a candle-light environment off-campus nor those with laptops could not put them to use due to power limitations.*

Such conditions are beyond individual student or institutions’ control. A proactive intervention was the national legislation, which required mobile providers to support ERTL through zero rating data (Chester 2020). National organisations Tertiary Education and Research Network (TENET) and Universities South Africa (USAF) negotiated with the providers regarding the technical process of ‘whitelisting’ educational sites. This is a particularly compelling case of sector level collaboration in order to overcome barriers to inclusion.

In addition to collaboration within and across the HE sector, there were numerous examples of family and community solution-making and generosity to overcome barriers. In one case, *a student was very excited to learn that the community in which she lives had pooled their funds and purchased a solar panel so that she can charge her laptop, when it arrives.* In another, parents of a student in a rural area with no access made personal sacrifices. A contributor shared that, *one student wrote to me today, informing me that a lack of response from the institution has forced the parents to rent her a room in the vicinity closer to the university. Since she needs to complete her studies in time, she wants to know if she is allowed to visit the premises to access network hotspots for connectivity.* Such responses are indicative of community-based and family investment in student success, and the commitment demonstrated to overcome barriers students face. They are also indicative of symptoms of what President Ramapohosa described as ‘a fundamental failing in our post-apartheid society’ (Chester 2020).

Institutions made plans to address such inequalities too. Drawing from their own finances, individual universities donated, loaned or financed devices for students. The options to do so were uneven, in terms of both the institutions’ budget affordances and the actual devices provided. Some of the solutions were inadequate, as *these only give access to the learning material and some of the online assessment options but are not suitable for typing fully fledged assignments that could be submitted electronically.* Similarly, those who supposedly had devices struggled with their limitations: *what does it mean to only have a smartphone for their studies? Or not even that? One of the lecturers called me today to ask how to deal with a student who only has access to a feature phone, and only in the evening, when the partner comes back from work.* It is clear that despite the well-meant actions of institutions, the question of access extends beyond simply access to devices and infrastructure, but to adequacy and quality.

The focus of the multi-modal approach was on students, but we were aware that it should not be assumed that all staff had devices suitable for teaching. One colleague spoke for many when writing that *this was a real eye-opener. Not only did many faculties not have laptops or desktops at home, but also no Wi-Fi, or the skills and competencies to engage with remote teaching (one would have suspected differently).* With the lack of devices at home, a significant barrier for staff, institutions made a plan: *due to context of the institutions (lack of enough funds), staff are also being allowed to take their desktops to use for teaching from home, with some providing loan machines or providing the machines with finance agreements.* It also emerged that institutions had different negotiating power and access to providers for coming to workable solutions.

Another problem arising from the solution of providing devices was that getting the devices to students across the country required effort beyond the usual scope of university practice, in addition to creating a range of risks, as highlighted within these excerpts. *The logistical task is just as complex as many delivery companies do not deliver to certain areas or are limited. Theft is another added challenge in this space—trucks with laptops being hijacked. But another challenge is delivering printed material or laptops to very rural areas. In these villages, houses are identified by the colour of the roof or house and there are no numbered houses or street names and parcels are normally dropped off at the local school, which are also now closed.* In one university, *the hard lockdown left many design students without access to their materials and equipment stored in lockers on campus. Lecturers sent all their students a standard kit as well as a smartphone on which all work could be done. These arrived by courier to local pick up points such as schools or shops in even the most far-flung corners of the country.* For some of the contributors it was a sobering experience to *realise the shocking lack of understanding (and resistance) on the part of the courier company and university middle-class staff of what a deep rural or township or ‘tribal authority’ area address for delivery should and can be.*

First power, then devices, then connectivity, then good quality content supported by interactive learning interactions—these emerged as the basics for access to remote online learning. This third issue is central, as for many connectivity is non-existent or of poor quality. An example is *a student was unable to download the 12 MB app for our LMS as he is located in an area with fluctuating connectivity; access is dependent on unequal connectivity.* To address this, providing data packages has been common. *Data was made available to students and lecturers by cell [phone] companies as well as the subsidising of data and devices by university management (also one of the surprises!).* But *all connectivity is also not equal. Lecturers and students experienced problems even with the connectivity that they had—some days it would work perfectly; other days it was abominably slow.* Even though sector organisations organised for the cell phone companies to zero-rate educational sites, this proved *not accessible to all given disparities in [mobile phone] connectivity across the country.* In some places it was really hard to access: *one of our students only had connectivity by climbing a tree.* Educators were further concerned as there are suspicions that the access for the zero-rated content was throttled.

Both staff and students have been imaginative in their methods of getting connectivity. In one case, where *staff do not have unlimited Wi-Fi at their disposal, nor the kind of connectivity one needs to engage in these synchronous engagements. I was told that the staff is sending each other data, so they can attend departmental or faculty meetings.* In another case, *especially near the clinic, you often see large groups of students trying to eavesdrop a wireless hotspot for Internet access.* This latter institution is of interest, because despite being located in a rural area comprising largely first-generation students on state-funding, student ingenuity led to a situation where *thus far the university has had 88% of the student population online.*

Lack of resources has been another risk. Because of the prompt restrictions imposed without notice, *some students left their textbooks in residence... when they left to be at the home towns during the lockdown period.* In many courses, *it now comes to attention that prescribed electronic books are not available.* To mitigate these risks, *many lecturers worked with librarians to distribute free resources and e-books to the students to assist them in gaining access to learning material.* South African copyright legislation, currently highly contested, was not helpful. The risk was also countered by the *opportunity to celebrate the openness, sharing and collaboration prevalent during the pandemic through the continued use of open educational resources.* This turn to the commons was a surprisingly rare comment, given the ethos and equity agendas of many of the contributors.

Whilst some institutions addressed the provision of connectivity and devices, thus mitigating against resource inequality, i.e. access to material resources in Therborn’s terms, the cultural capital that needed to accompany the move to ERTL, remained a risk.

## The Biggest Threat?

Material resources are clearly necessary, but even at this early stage, we realised that they were insufficient. *The insertion of emergency remote teaching supported by the provisioning of digital access does not guarantee immediate digital fluency. The assumption of digital fluency, for both staff and students, in the absence of habituated use, may be an oversight introducing an additional barrier to equity.* This was recognised *as the biggest threat.* Across the board, *different student levels of digital literacies are a concern... where it is expected of students to participate virtually in all learning tasks during an uncertain time period.* It became obvious that *the acquisition of such skills is difficult to fully pursue when one is already under pressure and [has] a sense of stress and uncertainty.*

Preparing students for undisclosed times of remote online learning was a challenge. *Student readiness is in question* is an oft-made statement. The plight, particularly of first-year students, arises in different ways. On the one hand, the self-discipline required by online learning is the focus. One contributor ascribed this to the transition to independent engagement of HE: *especially first-year students, fresh from the school system, could not adapt in time to the new university culture of teaching and learning. They come from a background where they were spoon-fed and now they must start to take control and responsibility for their own learning.* Another felt this was due to prior ICT learning experience from formal education and from digital cultures and affordances at home: *a large portion of the first-year student population come from previous[ly] disadvantaged backgrounds. They have not been subjected to any forms of ICT in the secondary sector. This disadvantages first-year students as interventions such as basic ICT literacy could not be fully implemented due to the pandemic.*

As discussed in the second theme, fully online teaching is rare and recent in South Africa. Therefore, *the students of those lecturers that were using the e-learning platform are now advantaged above those students whose lecturers fell behind in utilising digital technology.* Furthermore, *lecturers who had never been exposed to online teaching and its methodologies are not going to become experts overnight. S/he might have been a good face-to-face lecturer, but this does not mean s/he will be good in online teaching. And this might lead to the decrease in the quality of teaching.* Hence, these questions: what does it mean to complete the year by any means possible? Who assesses quality? What if we do not meet required pass rates? Will students be pushed through? Does it matter?

Finally, cultural capital pertains to motivation, the affect, and beliefs as well as to capabilities. With regard to ERTL, *obligatory expectations related to designing educationally sound online courses for an undisclosed time period could potentially highlight the divide between those who embrace technology and online learning and those that made a concerted choice based on epistemological or personal beliefs not to fully engage with online practices prior to the emergency period. Questions related to academic agency, but also the rights of such academics’ students form the basis of interesting debates about equal access and freedom of choice.*

Responding to the risk of digital fluency, cultural capital and inadequate resources were particularly challenging since students and staff were not located on campus or centralised spaces that would allow ease of access and provision of support. Instead, they have had to contend with living and learning spaces that presented a myriad of other challenges and risks that required university staff to respond differently, yet urgently.

## Places of Learning

Thrust into the world of online and distance education, it immediately became clear that the slogan of *anywhere, anytime, anyplace is a brutal underestimation of the complexities and entanglement of different inequalities and structural arrangements.* Overnight, *gone is the relative safe haven of campus life, social life and peer groups.* Students and staff were thrust into *a lack of dedicated space to work undisturbed and the need to care for family members and especially children who must be home-schooled during the lockdown.* Students reported *more family responsibilities like running errands, household chores, taking care of elderly family members*. Such role conflict emerged in *stories of students being admonished for being lazy and just reading (rather than physically active); for having even more pressure to choose between prioritising their time/finances for personal gain (their studies) or their families financial or care-giving needs.* For some, returning home meant returning to places of violence while residential accommodation on campus was *a refuge for those coming from abusive/dysfunctional homes—physical emotional and verbal abuse/gender-based violence.*

*While the university may offer inclusive teaching and learning programmes, some of the students’ homes are not conducive for learning. Some of the students stay in homes which are not suitable for studying. ‘Places of learning’ for our student cohort differ extensively. Large proportions of the student cohort have the privilege of a home environment that provides opportunity for continuous engagement with studies. However, many students live in circumstances which are not conducive for long periods of study and engagement with learning materials.*

For many students, access to campus resources, even if limited, is essential for pursuing their studies: *whereas students have various options on campus, such as learning hubs, informal meeting places or the library, many students are currently limited to areas which do not provide access to sufficient places for learning to take place.* Hard-won equity-driven initiatives to level the playing field on campus terrain disappeared overnight: campus computer labs, residence rooms, free Wi-Fi, all gone. *Parity has eroded as students for instance, no longer have access to computer labs.*

In a pandemic, everyone is affected: academics, students, as well professional and administrative staff. In addition, *the agency to change the situation is removed by the lockdown and the closure of the campus space.* Yet, staff and students are unevenly impacted and have to make substantially different sacrifices depending on their circumstances. For students and educators who are also parents, there has been a double burden that was most often born by women, as has been noticed in other contexts. This example no doubt represents the situation experienced by many: *there was one lecturer who can only pay her full attention to her work at night as she had to home school her little children and take care of other home related tasks during the day.* This extended beyond those teaching, with situations where *admin staff without laptops, staff without computers, many without Wi-Fi, many sharing devices with family, many with extended family in small surroundings [are] unable to ‘perform as professional’ within those spaces.* Staff and students without access to appropriate space and equipment were unable to perform their duties.

Notions of place, of learning environments, of the university itself have been disturbed during the pandemic, highlighting the entanglement and the socio-material nature of ERTL itself (considerations studied by Gunter, Raghuram, Breines, and Prinsloo 2020).

The daunting challenges discussed in the themes above were confronted to varying degrees by the time of writing this text in mid-July 2020. Responses were offered, agency was exercised where possible and numerous efforts were made in good faith. What we have recognised as particular risks, are the pedagogical aspects and implications on equitable academic success for all students.

## Parity of Pedagogy

Resource inequalities impacted directly on pedagogical design in ways with which residential universities were unfamiliar. The one exception has been the country’s large distance education university where the ‘pivot’ was not to different modes of teaching but rather to *summative assessment online, as well as how to move the administrative, ICT, finances and student support to remote working conditions.*

Without time and expertise for considered fully online design, and mindful that it was emergency remote teaching rather than ‘the real deal’ (Hodges et al. 2020), we were of the mind that it was essential that pandemic pedagogy (Williamson, Eynon, and Potter 2020) be designed as parity pedagogy from the outset.

With this in mind, attempts were made to address various inequalities simultaneously. *Most importantly, there is the pedagogical challenge: the rapid move from face to face to emergency remote teaching has stumped some academics and students.* Academic staff developers and other support staff have been trying to counter their colleagues’ panic and to articulate *the difference between online learning and our current state of emergency remote teaching.* Inequities are severe when pedagogical choices do not enable parity of participation, the definition of social justice held by Fraser (2007).

Contributors observed how synchronous tools and forms of interaction became practical, pedagogical and political matters. Pedagogical synchronicity was perceived by some students as creating much needed social presence and support. *Many students request synchronous lectures in order to experience a sense of engagement with the lecturer and their peers, yet this can be to the exclusion of those who do not have the necessary connectivity.* Interestingly, the virtual nature of real-time lectures was also considered advantageous as *some students commented on the fact that live virtual teaching sessions removed the distance between them and the lecturer. They remarked that the large classes in which they are taught created greater distance to the lecturer, than seeing the lecturer on their computer screen ‘as if talking just to me, personally’.*

The harsh practical realities of limits to connectivity, discussed in the theme ‘The basics’ above, have overall, however, promoted asynchronous and data light design, as this typical comment explains: *in our university’s case, the use of live online sessions to replace contact classes led to unfortunate exclusion of students who do not have enough data to pay for these sessions. Students could access recordings of these sessions without cost but could not participate in the live sessions.* This was because *none of the online video conferencing software will be zero-rated, so synchronous learning will have to be kept to a minimum.*

We have noticed that there has also been appreciation expressed for some of the pedagogical affordances of online learning: *students also commented on the usefulness of reviewing lecture recordings or narrated PowerPoints if they ‘lost track’ or did not understand a concept... The use of discussion boards was also mentioned by students as a useful communication channel that provided them more opportunity to engage with the lecturer than they would normally have during contact sessions. They can ask questions and receive answers asynchronously, allowing them to use their night-time data to participate.* Some of the data provided with panache by the cell phone companies was free only between midnight and 5 am.

Designing for low tech environments as a first principle means that, *what is much more complex to achieve is to create learning experiences that would allow student engagement with content, the lecturers and each other asynchronously. The kind of social learning that happens in the classroom—to recreate that using low tech tools and platforms—will be the real challenge for our staff.*

Despite the acknowledgement that, by its very nature, hurried emergency remote teaching could not deliver everything that carefully planned and expertly designed courses could, educators and professionals were, from the outset, aware of the importance of learning design for success and parity. The commitment to equity, as ‘access for success’ within the curriculum, is mainstreamed within the principles valued within South African HE’s teaching culture.

Expert learning design considers students’ contexts, where *equity is recognising that students are not homogenous.* Without learning design, there is a fear, that a hurried shift online would replicate ‘chalk and talk’ methods, where only *the lecturer’s hegemonic understandings dominate* and which would result in *moving bad teaching online creat[ing] a bigger threat to the success of these students who are not supported in elitist cultures with unequal power relations. Bad teaching perpetuates inequality.* Indeed, *as in traditional face-to-face environments, who is seen, who speaks, who is heard - these are worries.* Existential inequalities pervade the virtual as they do the physical. Notions of a collective responsibility (staff and students) added to the complexity of these challenges, *speaking to the notion of humanising pedagogies that came more to the fore during the time of ERTL. An emphasis is placed on the human aspects of online learning where human connection and reciprocal empathy are prioritised.*

ERTL forced staff and academic developers alike to think differently about assessment and established assessment practices. At the heart of concerns about equitable provision online is assessment, which proved *one of the biggest barriers is the implementation of online assessments and assignments.* Indeed, assessment methods are *causing unnecessary stress which might negatively impact on students’ performance.* For some this seemed insoluble: *currently, our institution is working on the scenario that students will come back to campus for summative assessment later in the year or even early in 2021.* Others noted the positives, recognising that *this has been an opportunity to rethink assessment, grading and related aspects* and *there is also much more flexibility around assessment and what could and should be submitted in various formats.* This, in turn, proved very demanding of staff as *that required quite an adjustment... we had five staff members who literally answered student queries 7 days a week.*

A concern is that special education needs (SEN) often took a backseat in the deliberations related to ERTL course design and implementation. *Courses are designed with a common denominator in mind with limited time provided to consider differentiation. So, students and staff members that live with disabilities are particularly vulnerable during a time of remote online learning.* Such students may also suffer the consequences of hurried learning design under pressure.

Across the world, there has been concern for disciplines difficult to teach online, one made more difficult in low-tech conditions. Innovative examples are emerging such as *studio based work being facilitated by means of self-made or open source demonstration videos, narrated PowerPoints and timed discussion sessions of apps such as WhatsApp.* As with so many other cases of problem-solving, *students and staff overcame many challenges through small innovations, grit and allowing many ‘redos’*. Academics joined and contributed to *global disciplinary networks, sharing ideas about how to teach design disciplines online*, another instance of collaborative resource-sharing but at an international level.

We have all observed how teaching and learning has come under the spotlight, revealing for example, *senior managers’ superficial knowledge of what is actually going on in teaching and learning*. And yet this has been *a massive student ‘success’ intervention experiment—never had we focused so much on students—students were called, emailed and invited to ask for help, and more students engaged with academics and their modules.* Furthermore, we are *heartened by the importance assigned to equity in most online discussions, webinars and conversations focusing on the pivot to emergency remote teaching and resulting in a general consensus regarding the adoption of low-tech and flexible teaching and learning options.* A student-centred approach which existed largely in phrases in mission statements is now central to decision-making, while equity approaches are clearly on the agenda. In addition, the composition of committees and formal structures to do with teaching and learning within institutions has seen changes to the inclusion of various student groups who previously were not included.

Going forward, there is an imperative to continue good practices. For instance, *the emphasis on the use of low-tech technologies to ensure that students with no or not enough data still participate in learning. This is something universities/or staff developers did not emphasise before the pandemic, but I think it should be something to consider in developing teaching and learning materials post the situation we are in.* While this concern at the level of pedagogy is important, what is needed are differentiated approaches and practices across education and disciplinary sectors.

## Sectoral Stratification

The shift to ERTL occurred in a sector where the South African government had already acknowledged that going online could be a risk. A recent report by the Presidency expressed the concern that, ‘a massive shift towards online education... could be detrimental to the South African education system’ (Presidency 2017: 547). The sectoral differentiation and stratification have been made visible. As a contributor expressed, *those of us speaking here work in very different institutions; urban/rural, highly/not ranked, low/high numbers of NSFAS (National Students Financial Aid Scheme) students, research/teaching focused. We recognise this existing stratification. It is likely that the current situation has shown the fact that there are still differences between the previously advantaged and disadvantaged universities. Some of the former [historically white] institutions are already teaching, while some of the later [historically black] are still grappling with how to implement.*

Existential inequality considers aspects of social capital, circles of influence, how one requests help, and one’s social circles and networks. From this perspective, some universities are ‘more unequal’ than others. *The historical context of an institution and the socio-economic profile(s) of its student population govern the response to this question. Our institution is located in the poorest province with the poorest Matric results in the country. Add to this that approximately 80% of the students are on state funded NSFAS bursaries.*

All universities found themselves in a crisis where they had to address inequities across their own student bodies, but the differences in the ability to respond and the needs of student bodies, have been exposed. On the one hand, a comprehensive response was possible by an historically advantaged institution: *our university used various means to remove barriers that could impact on students’ ability to continue their studies. The university used learning analytics, surveys, personal phone calls and other avenues to provide students with the opportunity to indicate the possible barriers regarding devices to continue with their studies. The number of students who needed assistance with a device were surprisingly small.* In another case, a different university *reached out to about a fifth of the total student population* to provide devices.

While all of the 15 universities whose staff contributed to this paper developed mechanisms to help prepare academics at speed for remote teaching, the capacity to do so was dramatically divergent. For instance, one of our universities had *the privilege of experienced, strong leadership and access to funding to support a multifaceted approach to academic support during a time of emergency remote teaching.* This included *several institution-wide approaches... faculty-based developmental opportunities... targeted online webinars that covered a wide variety of topics... an institutional staff support site with access to all the webinar recordings to assist them further… faculty-level blended learning coordinators and embedded, collaborative practices.* This contrasted with another of our universities where, *in the first 2 weeks of the lockdown, we developed a course to train the lecturers in the very basics on how to create the necessary learning material. Many lecturers completed the module and applied it with success. However, there are still some lecturers that have not completed the basic training.*

Even at highly ranked universities perceived to be well-off, there has been an enormous sense of frustration expressed by the staff and students in their midst. One wrote about how *this experience has emphasised our students’ very difficult circumstances and surfaced just how little we can provide in that regard while at the same time being very aware that the academic project needs to continue. Given our university’s capacity, realistically there is nothing we can do to change those circumstances.* The responsibilities of universities for social inequalities are firmly brought into question as well as how to respond to this demanding situation in the future.

## The Responsibility of Saving Lives, Saving Life Chances

The situation at the outset of the pandemic in South Africa has, and continues to be, one of horror and loss, of economic crises that will surely damage HE, of many profound risks to the equity agenda and of seemingly intractable conditions. Covid-19 has shattered the ivory tower as the boundary between the university and society has become manifestly porous with structural inequalities of the country laid bare. Indeed, *we are teaching from within the community we are living in right now.*

A recurring issue in HE debates is the extent to which universities can, and should, address social and economic inequities. *Equity in [higher] education is, to a large extent, about levelling the playground for all students to succeed... Access to quality education, food security, housing, healthcare and wealth creation is still very much limited for the majority of citizens in South Africa including those who enter into higher education.* Levelling the playing fields remains a huge challenge, nearly 30 years post-apartheid and the structural inequalities of its policies. We acknowledge that this is *a tall order for universities to deal with societal inequalities but that does not excuse universities from playing their part.*

Central to all our concerns is care for students, with the shift to ERTL making us *question the role of universities and the perceived responsibilities of universities... This made me think—how do students then understand the purpose of the university and what they are required to provide for all students?* In particular, we have observed how students have turned to the university expecting support: *it is been surprising to me to see the dependency students have on institutions and also the expectations they have of institutions.*

The question of who bears the responsibility for addressing the numerous challenges faced during this period has been a compelling one. *It was interesting to see that when the time came for online learning, students and beyond looked to institutions to provide solutions rather than the government. I was also surprised by the lack of national guidance for higher education.* Many of us wondered, *what then is the role of the government?*

In those circumstances of urgency, need and commitment to access, it emerged that individual institutions financed the numerous prongs of the multimodal strategies, not the state. There is sectoral agreement that these initiatives should continue because *nothing remains business as usual in the ‘new normal’ teaching and learning practices.* But *although mechanisms are put into place to balance the equity in teaching and learning, the danger to this is how long will the university be able to provide... This has huge cost implications for the institution, which were not budgeted for as the implementation of various ICT projects during the pandemic has cost [universities] substantial amounts of money. There is a need for resources for the postimplementation and support on the various ICT interventions [and] the revenue to sustain the projects.* It is alarming that, in a pandemic that has necessitated a shift to ERTL, underfunded universities already tasked with ‘saving money’ have been additionally burdened with the critical imperatives of using their scant resources to save lives and, using Therborn’s terminology, saving life chances. We believe that *universities working in collaboration with the government, [will secure] strategies for continued provision of the above items… post the pandemic.*

Against the tide of the pivot online globally, we are simultaneously distrustful of the *allure of market promises and the commercial entities and venture capital which have raced to provide ‘solutions’ for educational institutions.*

Individual universities have dug deep and individual educational actors have achieved astonishing feats. Yet, while it is important to acknowledge the commitment and sacrifices of the individuals who make up universities—students, guardians, lecturers, learning professionals and administrators—portraying individuals as heroes often serves the ends of letting those accountable for addressing structural failures off the hook. When considering accountability, we also ask questions about sustainability and systemic responsibilities. This is what White and Tronto (2004) call the duty of public care.

## Conclusion: on Repair and Re-creation

Now that the pandemic has put equity and inequality so indisputably on the higher education agenda, it has become evident, as so many have observed, that *nothing remains business as usual*. The pandemic has ironically provided possibilities for policy reformulations as well as for entrenching new practices that foreground flexible and equitable forms of provision. It has brought into focus numerous examples of extraordinary resilience, networks and at times unexpected alliances of collaboration and support, including inspiring creativity, examples of technology used for equity purposes and moments of optimism. In contrast to the entrepreneurial hype of Covid-19 EdTech companies’ innovation speak, there is an opportunity in the moment for genuine equity-focused innovation, policymaking, provision and pedagogy.

In the face of terrible loss and the serious risk of educational life chances with concurrent inequalities (vital, resource and existential), there are glimmerings of hope. We are *heartened by the fundamental concern for the well-being of all and the resilience to continue despite the overwhelming challenges, embracing change, no matter how uncomfortable it feels to be teaching at what appears to be ‘the edge of chaos’.*

What our reflections have shown is that the teaching and learning project is relational, and that none of these complex problems can be effectively solved by one autonomous component but that we are in Tronto’s (2001) words, entangled in a complex web of relations. In a caring relationship or web of relationships, no one person or group can be solely responsible for decision-making: all the parties at all levels of universities and of society should contribute to the discussion on caring needs and how they should be met (Tronto 2001). It is only by taking all stakeholders into consideration that ethical and caring conditions can be created. However, such deliberations and negotiations need time and trust, two resources that have been scarce in these times of crisis and in the terrain of historic and current inequalities in the country.

Still there are instances where staff within and across the institutions, in close collaboration with students and communities, have found ways to engage in an ethic of care—listening and responding to the needs of all parties involved. Through this, we have found innovative and creative ways of dealing with the crisis thus far. Ethical practices come down to everyday decisions of care in caring relationships (Tronto 1993; 2001), and in this paper, we have attempted to have a closer look at these everyday decisions that are taken by all teaching actors together with students and their communities. These intersecting, interwoven conversations are in some ways an ethical practice. What we have seen repeatedly in the recent past, is that if institutional care and support are not in place, lecturers and learning professionals step in to form relationships and communities of practice to facilitate self-care and care for others—to be both caregivers and care receivers. In contrast to the individualistic profit-motivated notions of care punted as educational remedies (see for example Williamson 2020), this ‘in-common based on the possibility of sharing unconditionally… is a thing uncountable, incalculable, priceless’ (Mbembe 2020).

During this period, fields of practice and scholarship, which had previously intersected far less than one would have imagined, are now thrust together. The scholarship has drawn on different theoretical sources. The practice has been supported institutionally in different ways, either centrally or distributed. Historically, questions of access to and success in education were the purview of ‘academic development’, while the digital divide and digital inequalities fell in the parallel realm of ‘educational technology’. These separations have been shown to be impossible, *with Student Affairs thrown into the mix as students demand that #NoStudentIsLeftBehind.*

The nexus of these transformational issues requires a new way of seeing and not unseeing what needs to remain visible. This is where the hope lies. The pandemic has been an MRI exposing the social bones (Roy 2020), an X-ray making it possible ‘to see all the broken places’ (Wright 2020). Thus, our reflections of ERTL in this paper illuminate multiple and coexisting forms of inequality in higher education. While this might seem hopeless at times, recognising care as repair embraces the notion that ‘when people [and indeed systems] confront their failures, they have the opportunity to mend them’ (Wright 2020). Clear analysis of the complex shape of the terrain is essential, as is resistance. Harder to grow, yet fundamental, are the seeds of community, collaboration and commitment which can restore and recreate a deeply damaged sector.

## A Final, Personal, Word on Hope

On a personal note, we are still hopeful, we cling to hope, although we know that this hope is fragile. It is also an angry hope, because we, as with many of our colleagues, are at the forefront of this pandemic and are dealing daily with the impact of the glaring inequalities our society and our institutions are steeped in. Hope sometimes feels wrong, in particular when we feel we are supporting a broken system to survive with our feeble attempts at saving the unsavable. Hope feels torn, because we are uncertain of what is right and what is wrong. Hope is dogged, because we nevertheless continue our work on a daily basis. Hope is resilient and collective because as communities, we do find ways to cope, but hope is also compromised because we know with every move we make to support some, we leave others behind. Hope is critical because we keep calling out systemic injustices, but hope is also insistent because it is impossible to give up as long as possibilities exist for equity-oriented change.

## Footnotes

1 So, early on, there was little time for recording and reflection. It is to be expected that analyses will come later. An early exception is one institutional account (Motala and Menon 2020).

2 Most of the authors were part of a national workshop in 2019 on considering a blended learning future in South African higher education. Some included other colleagues in their reflections, some were unable to contribute, and some had not yet shifted online at the time of writing. Not all contributors elected to be named in this paper.
